# News from Biology Open in 2020

**DOI:** 10.1242/bio.051821

**Published:** 2020-04-03

**Authors:** Rachel Hackett

**Affiliations:** The Company of Biologists, Bidder Building, Station Road, Cambridge CB24 9LF, UK

Fears that a long-planned meeting of the Biology Open (BiO) Editor team would be disrupted by the then-scheduled Brexit were unfounded, with the team from the BiO editorial office and the majority of BiO's Editors gathering in Oxford on Halloween 2019. This was the first Editor meeting hosted by current Editor-in-Chief Steven Kelly, who treated the Editors to a tour of his college. The meeting itself involved an in-depth review of BiO's progress, and a lengthy discussion of how to meet the challenges faced by BiO in the ever-evolving publishing ecosystem (e.g. the implications of Plan S, becoming an affiliate journal for Review Commons).

**Figure:**
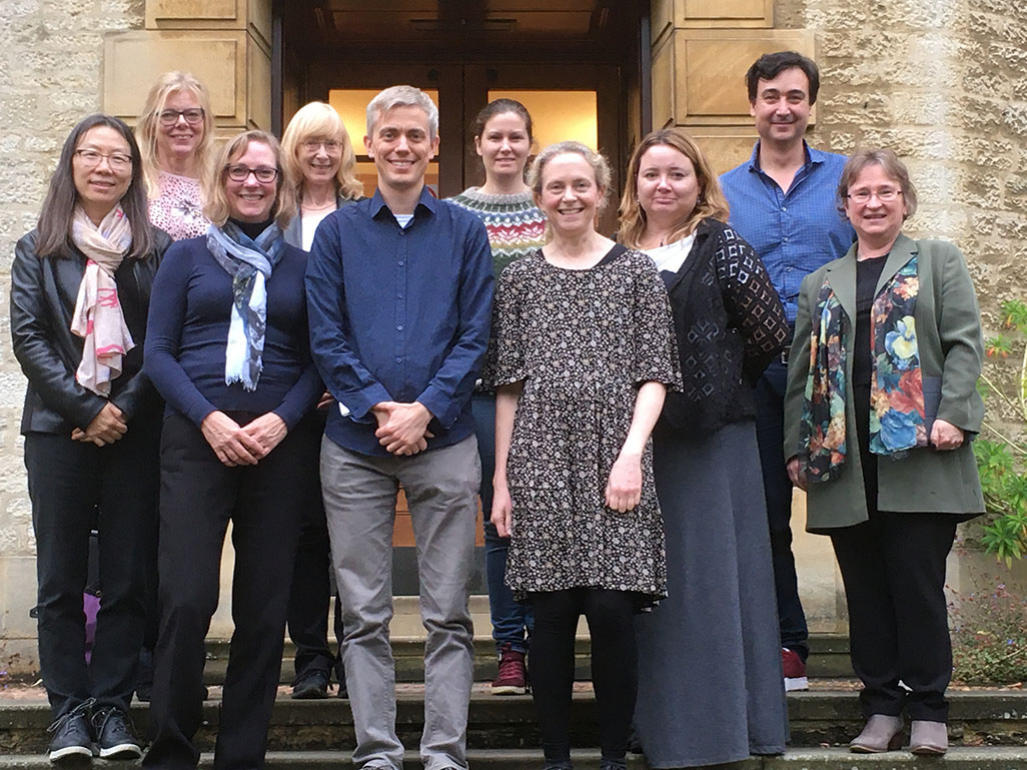
BiO Editor meeting. Yishi Jin (Editor), Sue Chamberlain (BiO Editorial Administrator), Kendra Greenlee (Editor), Cathy Jackson (Editor), Steve Kelly (EiC), Sjannie Lefevre (Editor), Laura Machesky (Director of The Company of Biologists), Rachel (Managing Editor BiO), Tristan Rodriguez (Editor) and O. Claire Moulton (Publisher). [Other Editors and Directors attended but are not in the picture.]

The Editors also heard from an early-career ambassador, Steven Burgess from the University of Illinois and previously eLife Editorial Community Manager. Steven touched on the involvement of early-career researchers (ECRs) in the review process, highlighting the importance and advantages of using ECRs and dispelling certain myths. The issue of ghost-writing of peer reviews by junior researchers was also mentioned (and has been highlighted elsewhere). BiO encourages authors and Editors to consider diversity in career stage, geographical location, gender and ethnicity when suggesting and selecting appropriate reviewers for a manuscript. [This was prompted, in part, by a gender analysis conducted across The Company of Biologists' journals (see Box 1)]. For ECRs to be involved in the peer review process, BiO requires that there must be a genuine mentoring process and the senior invited reviewer should always take final responsibility for the report delivered to BiO. The name of the co-reviewer must be reported to the Editor and a field is provided in the report form for this purpose. The names of these co-reviewers are also included in our annual published list of reviewers (see supplementary material). We thank every one of them for their expertise and time, as well as our authors, readers and editors for their support.

**Box 1. Gender analysis across The Company of Biologists' journals.**

This analysis was done primarily by Sam Holden, a PhD student at The Sainsbury Laboratory and University of East Anglia, Norwich, who in 2018 spent three months as a Professional Internships for PhD Students (PIPS) intern with us as part of his PhD program. Sam was helped in his analysis by the Royal Society of Chemistry (RSC), who developed the algorithm used to assign gender to names (and have recently published detailed statistics on gender bias: https://www.rsc.org/globalassets/04-campaigning-outreach/campaigning/gender-bias/gender-bias-report-final.pdf). Many thanks to Sam for his work on this project, and to our colleagues at the RSC for their support.

**Methodology**

For each of our five journals (Development, Journal of Cell Science, Journal of Experimental Biology, Disease Models & Mechanisms and Biology Open), we downloaded data on all research papers submitted between October 2006 and May 2018, and extracted the following information:

• Outcome of submission (editorially rejected, rejected post-review or accepted)

• Name of first author

• Name of corresponding author (note that this may be the same as the first author)

• Names of individuals suggested by authors as potential reviewers

• Names of individuals invited to review the paper

• Names of reviewers who completed a report on the paper

We ran the lists of names through an algorithm that assigns gender to names, along with a confidence value in the assignment. We assigned a gender to names where the confidence value was greater than 90%, allowing us to assign gender to ∼75% of authors and 85% of reviewers. It should be noted that the algorithm was developed using a dataset of mainly Western names, and the majority of names with ‘unassigned’ gender are Asian. Thus, the results outlined below do not necessarily reflect patterns that might apply to non-Western authors and reviewers.

To allow more rigorous statistical analysis, data were pooled across all the journals and the whole >10-year time span, although we have also looked at trends over time and between journals.

In addition to calculating basic statistics on the gender balance of our author and reviewer pool, we also analysed the success rate of submissions based on author and reviewer gender.

**Key results** (combined data for all five Company journals)

• Almost exactly 50% of first authors (typically the junior researchers who contributed most to the research) are female – implying minimal gender disparity at the level of the PhD students and postdocs in our community of authors. However, among corresponding authors (typically principal investigators/lab heads) only 30.3% were female.

• The gender of the first author had no influence on the success rate of the submission. However, papers from female corresponding authors showed a slight, but statistically significant (*P*<0.05), reduction in acceptance rate – only 28.5% of corresponding authors on accepted papers were female.

• Disparity is seen at both initial editorial assessment and at peer review: papers with female corresponding authors are less likely to be sent out for peer review than those with male corresponding authors (67.3% vs 71.0%) and, once sent out for peer review, are less likely to be accepted for publication (52.9% vs 56.2%).

• There is a greater gender imbalance in our pool of reviewers than in our pool of corresponding authors: 26.1% of people invited to review a paper are female and 25.8% of completed reviews are by women (the similar numbers suggesting that both genders are equally likely to accept an invitation to review). These figures have improved over the 10-year time window: in 2007, only 23% of reviewers were female; this reached 29% by 2017 (though this is still below the 30% proportion of female corresponding authors).

• Authors are more likely to suggest reviewers of the same gender as themselves. However, we have not found evidence that female-authored papers are at a disadvantage if reviewed by men (although the data on correlations between author and reviewer gender are hard to interpret).

This box has also been reproduced in other Company journals. See also an Editorial (Briscoe and Brown, 2020) from the Development team that explores the subject in greater depth.

BiO is also supporting its reviewers through a trial integration of its online peer review platform with Publons. Reviewers can now choose to add their BiO review to their Publons profile when completing the reviewer form (via an automated process). The profile can then be used in job, visa and grant applications, complete with journal-verified review activities.

The BiO Editors were also the first to hear about ‘BiO Meeting Reviews’ – a new initiative from BiO and its publisher – the not-for-profit Company of Biologists. We are delighted to announce that recipients of Scientific Meeting Grants from the Company will be eligible to apply to publish a Meeting Review in BiO free of charge. These Meeting Reviews will summarise the emergent themes and discussions, and provide a platform to support dissemination and access to meeting content for the global biological sciences community. The first two examples of such Meeting Reviews are already published (Raina et al., 2018; Reynolds, 2018). Detailed information about the application process will be provided to meeting grant recipients, and more information can be found at https://bio.biologists.org/meetingreview. BiO is fully Open Access (articles are published under a CC-BY license), has the DOAJ seal and is indexed in PMC, PubMed, GoOA, Scopus and Web of Science. BiO Meeting Reviews are an excellent way to increase the dissemination goals of your meeting, and ensure free global access and maximum visibility to the scientific community.
